# First Aid for the Cinema "Artist"

**Published:** 1923-09

**Authors:** 


### FIRST AID FOR THE CINEMA "ARTIST."

When you watch the charge of a cavalry unit, the swoop of the perilous aeroplane, or a rescue scene as it flashes across the screen, have you wondered at the aftermath ? asks Mr. T. B. Brownfield, of Los Angeles, in *The Trained Nurse and Hospital Review* of New York. Have you, he continues, thought that behind the scenes, doctors and nurses must be ready to handle accidents, and that nurses have tested the pulse and referred special actors to physicians before they attempted their hair-raising "stunts" ? Perhaps not. Yet behind the elaborate set, the Kleig lights and the strain stands Mrs. Randall at the Metro Studios in Hollywood; two doctors are also on call at all hours.

### "The Four Horsemen of the Apocalypse."

Not long ago, an actor, playing the part of a minister, was ascending steps in a thrilling scene where "the fires of hell" were licking up from below. Losing his balance, the player fell into a chute a distance below and was badly injured. Taken at once to the emergency clinic, he was given first-aid treatment, and then removed to a local hospital, where he is now recovering from a badly fractured vertebra. This accident occurred "on the lot," which, in "movie" parlance, means within the confines of the plant. But many accidents occur "out on location" (away from the studio). Again, the emergency clinic plays an important part. In the filming of "The Four Horsemen of the Apocalypse," for example, a field hospital was installed when a doctor and nurse were kept busy for five days on all day duty. This was necessitated by "wartime" scenes. Usually first-aid kits, prepared at the Studio's emergency hospital, are sufficient to meet the needs.

### The Cinema in the Field.

Each time a company goes out "on location" —and there are several companies producing at the Metro Studios—Mrs. Randall sends first-aid kits containing the usual necessities such as gauze, bandages, cotton, adhesive tape, splints, arm slings, tourniquets, scissors, forceps, solutions for burns, aromatic spirits of ammonia, iodine, aspirin, carbolated vaseline and some disinfectant such as lysol. Among each group of people sent out, one or two members know how to administer first-aid treatment. Other kits are distributed at various places "on the lot" where they are available for instant use.

### Looking After the Staff.

But the real work of the clinic *personnel* is to take care of the hundreds of workers at the studio, about five hundred daily. Many of these workers are called "grips," a term applied to the men who do the odd jobs about the studio, move scenery, and place "properties." Usually the cases treated at the hospital comprise small lacerations or foreign bodies in the eye, especially sawdust in the carpentry shop or splinters of glass when pieces are ground. The "grips" are constantly running nails into their feet; the electricians may be burned in handling their special line of work. "Kleig eye," a distinctly "movie" condition, evidenced in temporary loss of sight due to exposure to the Kleig lights, is treated with saline solution. The cases are usually mild. The emergency hospital is open as long as there are workers at the studio; and this may be far into the night or all night, for motion picture production often means twenty-four hour "jobs," the men working under rush orders all night, to get the "sets" ready for filming next day. From eight-thirty in the morning until five in the afternoon, the nurse is on duty. After hours the supplies are left where they may be available for instant use.

### WAITING FOR MR. SPAHLINGER.

Further publicity has been given to the Spahlinger treatment of tuberculosis by the discussion in the House of Commons just before Parliament rose. The position of the Minister of Health in the matter has been quite clearly stated, and seems to us unexceptionable. He is most anxious to encourage further trial of the new remedy under expert observation in this country as soon as a supply of the complete serum and vaccine is available. The clinical results already obtained establish a *prima facie* case for further scientific enquiry. Such an enquiry must precede and justify any expenditure of public money. We hope that Mr. Spahlinger will take early advantage of the opportunity which is being afforded him of making a full statement of his process and results to a body competent to pass expert opinion upon it. In the meantime we feel sure that the appeal which is being made for private funds to assist Mr. Spahlinger in the research on which he is understood already to have spent a private fortune will meet with a generous response.

